# Tonal properties in a non-tonal language: The case of Indonesian

**DOI:** 10.1016/j.heliyon.2023.e13440

**Published:** 2023-02-02

**Authors:** I Nyoman Udayana, I Nyoman Aryawibawa, I Nyoman Sedeng, Joan A. Sereno

**Affiliations:** a Faculty of Humanities, Udayana University, Indonesia; b College of Liberal Arts and Sciences, Kansas University, USA

**Keywords:** Tonal properties, Pragmatic meaning, Intonational expression, Interrogative contour

## Abstract

This study investigates the tonal properties of Indonesian, specifically examining intonation contours in Indonesian at the sentence level and focusing on how the tonal system is used to indicate different pragmatic meanings in Indonesian. Four distinct intonation contours with four distinct meanings were contrasted: strong agreement to the truth of the relevant word (emphasizing) (H), interrogative meaning (LH), doubting the fact of the word in question (L), and a neutral conceptual meaning of the relevant word (HL). The stimuli for the study were four Indonesian words (two verbs *makan* ‘eat’ and *tahu* ‘know’ and two adjectives *cantik* ‘beautiful’ and *marah* ‘angry’). Stimuli were recorded to capture the four-way contrast in pitch contour. A comprehension task to identify the distinct meanings was conducted. Forty-nine participants were asked to listen to the four words with the four different pitch contours and select their respective meanings. The data show that the participants were able to apply the Mandarin four-way contrast pitch contour to Indonesian to accurately indicate four different (pragmatic) meanings. The most difficult contour for the listeners/participants was in distinguishing the interrogative intonation contour (LH) from the low-dipping intonation contour (L) signaling doubting the conceptual truth of the lexical items used. The overall study suggests that a tonal system based on Mandarin tonal contrasts can be applied to Indonesian intonational expressions.

## 1 Introduction

Indonesian is a language characterized by the use of melody in a sentence, reflected by rising and falling, and level intonation contours. These intonation contours operate within an intonation phrase (IP), corresponding to a clause at the syntactic level [[1], [2], [3]].

At a sentence level, Indonesian intonation can fall on a sentential element such as Topic (T), subject (S), or predicate (P), with word order flexibility of the clausal constituent allowing for variations of the intonational contours as shown in Refs. [4,5]. Given that the intonational contours carry meanings with them, the variation permits speakers to convey different (pragmatic) meanings. The differences in meaning depending on the intonation structure can create distinct emotional or attitudinal meanings [[6], [7], [8], [9]].

At a word level in Indonesian, in line with Stoel [3] in his account of Manado Malay, a prosodic constituent referred to as a phonological phrase (PhP) contains one or more phonological phrases. An IP typically does not contain PhPs but is instead characterized by a pause break to indicate the different intonation and an intonation boundary to indicate a new sentence; hence, it ensures the occurrence of a new IP structure in discourse. What is interesting is that a word in Indonesian may represent a clause or an elliptical clause that only contains one utterance and it may carry with itself tonal properties which resemble the feature of lexical tones yet maintain its function as an intonational expression with distinct meanings (dependent on the preceding sentence or context in which they occur).

Historically, the Mandarin tonal system has incorporated its way into Indonesian through contact between the two languages. However, such language context has not resulted in the adoption of lexical tone in the Indonesian language. Contact between Mandarin and the local Indonesian languages can be traced to the influx of Chinese immigrants coming to the Indonesian archipelago. What is noted in the literature concerning the contact is that it began with the emigration of Fujian people from the southern part of China to Indonesia starting as early as East Han Dynasty (AD 25–220) up to the Qing Dynasty (1616–1911) [10]. Zhi claims that, out of the eight Malay-Indonesian dictionaries that he studied, 89% of Chinese loanwords come from Fujian versions of Chinese. The evidence for lexical borrowing from Mandarin Chinese can be shown by lexicons having to do with culinary terms, trade, and common items. In addition to this extensive borrowing, another variety of Chinese that has significant speakers in Indonesia is the Teochew language found in Jambi and West Kalimantan which are respectively called Jambi Teochew and Pontianak Teochew [11]. Indeed, overseas Chinese living in Indonesia do not only settle in the larger islands such as Java, Sumatra, Sulawesi, and Kalimantan but also spread into other parts of Indonesia predictably resulting in a focused interaction between Chinese and the native Indonesian population. The borrowings might include not only words but also the pitch pattern which is applied to the Indonesian intonational patterning since Mandarin acts as a substrate language in some domains.

In tone languages, distinct lexical pitch contours represent different meanings in the lexicon. This can be illustrated in Mandarin Chinese, a tone language, in which four segmentally identical words have four different pitch contours identified as T1, T2, T3, and T4 respectively (see Table 1), resulting in 4 distinct lexical items with 4 different meanings [12,13].

**Table 1:** Mandarin tonal system.

Pitch Shape	Example
T1 High Level (H)	mā ‘mother’
T2 Rising (LH)	má ‘hemp’
T3 Low or falling-rising (L)	mă ‘horse’
T4 Falling (HL)	mà ‘scold’

The phonemic tonal differences result in 4 distinct meanings attributable to the different tones. Mandarin Chinese is thus labeled as a tone language.

Tone and intonation relate to prosody (i.e. phonetic features occurring at a higher level of an utterance) and they share the same characteristics in the use of pitch [12,14]. In intonation (languages), the pitch distinction commonly operates post-lexically. It thus conveys information about the syntactic constituents of an utterance. In tone (languages), however, the pitch distinction is used within a word. It conveys information about the meaning, grammatical function, or morphology of the associated words. The use of pitch in intonation might extend to operating within a word. However, it must be borne in mind that in such usage, the associated words retain their core meanings but exhibit nuance differences related to pragmatically motivated interpretations as highlighted in Ref. [[14], [12]]. This phenomenon is more explicitly discussed in Ref. [13]. This again confirms that a language using this distinctive pitch maintains its status as an intonation language; it does not change its status into a tonal language.

Interestingly, nontonal languages such as English can also exhibit lexical pitch distinctions. The study conducted by Ref. [13] demonstrated how pragmatic meanings in English are affected by distinct pitch contours, as illustrated in (1).

**Table:** 

(1)	HL	LH
	cat	cat
	(neutral)	(question)
		[13]

In neutral intonation contexts, that is, in a declarative clause environment, the word *cat* has a falling (HL) pitch contour whereas, in question intonation contexts, the word *cat* has its rising (LH) pitch contour. The examples in (1) do show that English intonation has differences in (pragmatic) meanings, i.e. neutral and interrogative meanings.

Numerous past studies dealt with Mandarin tonal systems [[15], [16], [17], [18], [19], [20]]. They all seem to agree that the perception of Mandarin tonal system by speakers of tonal languages and in comparison with those of non-tonal languages specifies that the speakers of tonal languages perform better at perceiving the properties of Mandarin tones than those of non-tonal language speakers. The reason for this is shown by the fact that in the former case, the speakers are already equipped with the knowledge of tones generally makes them exert less or no effort in capturing the tonal features amounting to the fact that they are more experienced and skillful in making use of the tonal system.

On the other hand [21,22], view that the comprehension of a sound system to some degree depends upon their listeners' linguistic experience and their native language sound system which also plays a role in their perception. This means that listeners’ knowledge of their native language phonological system contributes equally to speech perception. The interplay between phonology and perception is therefore a two-way process, rather than the view that perception treats phonology as a unidirectional process. This situation can be exemplified by the fact that the Thai listeners might use Thai internal properties of the tonal system that share some similarities to the Mandarin tonal system to perceive the Mandarin tonal system itself [23].

The past (perception) studies on the Indonesian intonational system only investigated intonation concerning pitch movement, i.e. falling and rising pitch and word order at a sentential level (e.g. Refs. [5,24]). No studies have been conducted thus far on the application of pitch-contour patterning to nontonal languages such as Indonesian. The research question of the study is as follows:

RQ: *Does Indonesian have tonal properties in its intonational patterning*?

The significance of this study is to uncover the properties of the Mandarin tonal system applied to the Indonesian intonational system. Our study on the tonal properties of the Indonesian intonational system was drawn on Duanmu's work [13] which claims that English has a clear example of the correspondence between tonal pitch shapes and intonational expressions in English on neutral intonation (corresponding to T4 in Mandarin) and intonation associated with an interrogative intonation (corresponding to T2 pitch shape in Mandarin). Based on this we have made further investigation and found out that Indonesian also possesses T1 and T3 pitch shapes to be applied in the Indonesian intonational pattern.

Given the historical connections between Indonesian and Mandarin and using the English intonation features as a point of departure, the current study is to find out whether Indonesian utterances, using the four-way contrast of the Mandarin tonal system, can uniquely map distinct pragmatic meanings in the Indonesian intonational structural system. To achieve the purpose, four target words are selected (two verbs and two adjectives), with four distinct pitch contours. One-word utterances are specifically chosen to show that these words may behave like words with lexical tones and, further, that they exhibit dedicated and distinct intonational expressions.

## 2 Method

### 2.1 Participants

Forty-nine students participated in this study. At the time of the experiment, the participants were fifth-semester students of the Bachelor of English Program, the Faculty of Humanities, Udayana University, comprising 12 males and 37 females whose average age was 20.5 years old. None of the participants had any knowledge of Mandarin. (All participants have some knowledge of English.) The participants came from a variety of local areas in Indonesia. Given that Indonesian speakers can also be speakers of many different local languages, our participants can be considered to be representative of the speakers of local languages within the Indonesian archipelago. Out of the 49 students, 30 people also speak Balinese, 6 of them also speak Javanese, 3 people also speak Sundanese, 2 Bima language, 3 Jakarta Indonesian, 2 Sumba language, 1 Riau Malay, 1 Jambi Malay, and 1 Sasaknese. Informed consent was obtained from all the participants. The method employed to recruit the participants in this study was the convenience sampling method [25]. This experiment reported on in this article had also obtained approval from Unit Komisi Etik Penelitian (the Research Ethics Commission) of Udayana University.

### 2.2 Stimuli

Four Indonesian words were used for the comprehension experiment. The stimulus words are two verbs (*makan* ‘eat’ and *tahu* ‘know’) and two adjectives (*cantik* ‘beautiful’ and *marah* ‘angry’).^1^ We give diacritics to the words in question to relate them with their respective pitch contours (e.g., *mākan*, *mákan*, *mǎkan*, *màkan*). All words from all lexical categories (also lexical categories other than verbs and adjectives; e.g. nouns, prepositions, adverbs) can be employed for the target words consistently yielding the same pragmatic meanings across all the pitch shapes (T1 to T4). For this experiment, we chose two verbs and two adjectives. It is also worth noting that we selected the verbs that are categorized into bare forms rather than those belonging to the inflected forms (such as *memakan* and *mengetahui*) for the verbs meaning ‘eat’ and ‘know’ respectively. The reason for this is that it is the bare verbal or informal forms of the verbs that are more often used in intonational expressions in Indonesian. The validity of the stimulus words was examined through a pilot experiment which was conducted one week before the actual experiment. Fourteen different students (then used in the main experiment) of the master program of linguistics, the Faculty of Humanities Udayana University participated in the pilot experiment. They were sent a Google form containing questions about the stimuli. The pilot experiment contained 14 questions and they completed the task in 15 min. The reliability of the data was obtained using the method of test-retest wherein the participants were asked for the second time one month after the actual test was conducted to take the same test, but this time the ordering of the question items was changed. The result found that there was a high correlation between the results of the first test and those of the second test (0.88).

To ensure that the four-way contrast of the lexical tones in Mandarin is applied in Indonesian, three students were recruited to practice the correct assignment of the different pitches associated with the stimulus words. The three students were seventh-semester students of the Bachelor of English Program who can speak English, Indonesian, and Balinese but they cannot speak Mandarin. The online practice was given by a Mandarin teacher from the Gapper International Volunteer Organization who speaks Mandarin and English but does not speak Indonesian.

The three students were recorded for the listening task material. Two of them took part in the recording associated with each stimulus word (producing emphasizing, interrogative, doubting, and neutral intonation pitch contours) and the other student read the questions associated with the contextual meaning of the stimulus words being asked about. The recording was done in a quiet room using an MPL player. The acoustics of each of the recorded target words were checked using the Praat software [26]. Initially, we recorded 6 words for the data. In addition to the four words (two verbs and two adjectives), we intended to use two pronouns, *dia* ‘third-person singular’ and *kamu* ‘second person’. However, the two words were not produced with distinct acoustic tonal cues. These two words were thus discarded. Only the four target words that matched the acoustic characteristics relating to the tonal patterning were used in the dialog for the comprehension task, totaling 16 distinct stimuli altogether. The recorded material that contained the task was saved in a Google form.

### 2.3 Acoustic contexts

To help the participants/listeners capture the meaning of each stimulus word, a context was provided. Given the four Mandarin pitch contours are tonal while those applied to Indonesian are intonational, for our purposes, the names of tones of the Mandarin system are adapted to the Indonesian intonation system based on the meaning of each intonational expression. Thus, the Mandarin tonal system that is associated with the Indonesian intonational system and its associated meaning can be specified as follows:

**Table:** 

(2)	T1 corresponds to emphasizing intonation (H) in Indonesian
	T2 corresponds to interrogative intonation (LH) in Indonesian
	T3 corresponds to doubting intonation (L) in Indonesian
	T4 corresponds to neutral intonation (HL) in Indonesian

**Fig. 1:**
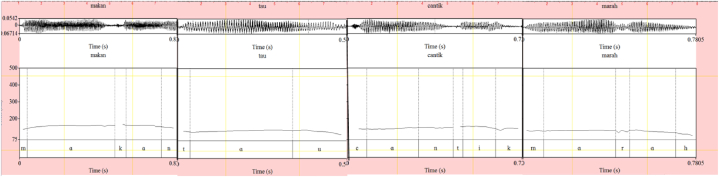
Emphasizing (.) (H) intonation for *makan* ‘eat’, *tahu* ‘know’, *cantik* ‘beautiful’, and *marah* ‘angry’.

**Fig. 2:**
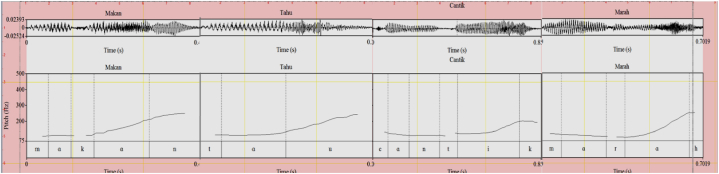
Interrogative (.) (LH) intonation for *makan* ‘eat’, *tahu* ‘know, *cantik* ‘beautiful’, and *marah* ‘angry’.

**Fig. 3:**
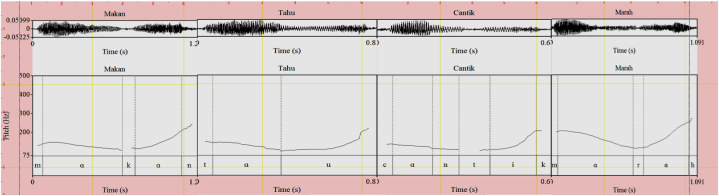
Doubting (.) (L) intonation for *makan* ‘eat’, *tahu* ‘know, *cantik* ‘beautiful’, and *marah* ‘angry’.

**Fig. 4:**
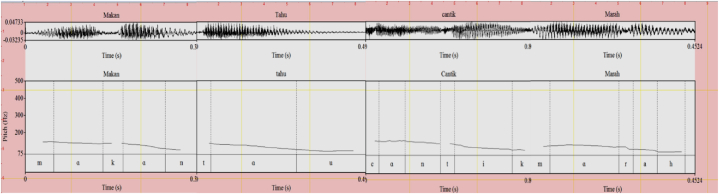
Neutral (.) (HL) intonation for *makan* ‘eat’, *tahu* ‘know, *cantik* ‘beautiful’, and *marah* ‘angry’.

The pitch contours denoting the acoustics of each intonational expression are respectively shown in Fig. 1 (emphasizing H), Fig. 2 (interrogative LH), Fig. 3 (doubting L), and Fig. 4 (neutral HL). The F0 for each acoustic segment of the stimuli is listed in the Appendix.

### 2.4 Procedure

A comprehension test was created for the participants to capture the meaning of the four-way contrast of the Indonesian intonational system. The recorded comprehension test material, which was saved in a Google form, consisted of 16 questions. For each question, participants were to listen to the intonation contours and decide the meaning of the utterance: emphasizing, interrogative, doubting, or neutral intonations. The recorded material was distributed to the participating students who already formed a WhatsApp Group. The participants were instructed to listen to the question by clicking the sound file. Then they were asked to answer the questions by selecting one of the four choices - the four-way contrast of the Indonesian tonal system. No intonational markers were provided in the question sheets nor were they (the intonational/tonal instructions) given to the participants. They were to select only one answer out of the four choices that they thought reflected the acoustic context they interpreted from the speakers uttering the intonational expressions. The instructions were all in Indonesian. They answered the task online at their respective homes. They were allowed 20 min to finish the task and it took them an average of 15 min to complete it.

## 3 Results

**Fig. 5:**
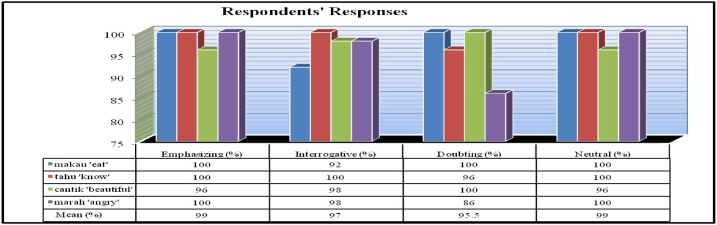
The percentage of the correct meaning choice of the target words.

The study used the comprehension task for the participants to capture the properties of the Mandarin tonal system applied to the Indonesian intonational expressions. The results of the comprehension task can be summarized in Fig. 5.

Overall, the 49 participants can accurately perceive the meaning associated with the target words (89% correct). For emphasizing expressions, the mean accuracy was 99%. For interrogative expressions, the mean accuracy was 97%. For doubting expressions, the mean accuracy was 95.5%. And for neutral expressions, the mean accuracy was 99%.

Each contour of the intonational expressions in Indonesian complies with that of the tonal system of Mandarin which can be depicted as follows. The emphasizing intonation is characterized by a level high pitch (H) on the syllabic nucleus. The interrogative intonation can be transcribed as having a low rising pitch (LH). The doubting intonation (L) is signaled by a falling and a rising or dipping pitch (L). And the neutral intonation is marked by a falling pitch (HL).

## 4 Discussion

A word in Indonesian, dependent on the preceding sentence or context in which it occurs, may represent an intonational expression with distinct meanings and they may carry tonal properties which resemble the feature of Mandarin lexical tones. The current study examined this issue. We created a comprehension experiment involving four Indonesian expressions with distinct pitch contours on two verbs and two adjectives. We found that Indonesian speakers can accurately identify four distinct pitch contours and map them onto distinct intonation meanings in Indonesian. We thus relate the possible internal phonological features of the Indonesian intonational expressions with Mandarin tonal shapes. The loaned pitch contours of the Mandarin tonal system can be applied to the Indonesian intonational system. In what follows, each Indonesian pitch contour and its associated meanings are discussed.

### 4.1 Emphasizing (H) intonation

Emphasizing is one of the most common intonational patterns used in Indonesian. In everyday life especially in a colloquial conversation, it is commonly used, as the name suggests, for emphasizing the meaning of the word in question. The nature of the emphasis is different from one lexical expression to another. The evidence for this is shown by similar phonetic features that Indonesian shares with the Mandarin T1 tonal feature. That is, the similarity to Indonesian is shown in expressions that we can refer to as indicating diminutive or augmentative meaning in expressions belonging to an adjective.

Consider the one-utterance *cantik* ‘beautiful’ in (3). The first syllabic nucleus of the word is realized by the open vowel/a/in the word *cantik* and has level intonation as seen in the pitch contour in Fig. 1. The associated meaning with this level intonation indicates that speaker B in (3) strongly emphasizes that Anita is very beautiful.

**Table:** 

(3)	A: Anita cantik
	Anita beautiful
	‘Anita is beautiful’
	B: Cāntik
	‘(Anita is) very beautiful’

**Fig. 6:**
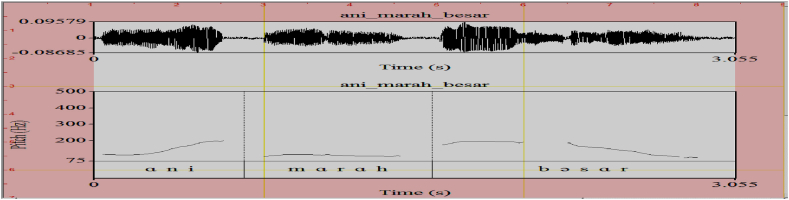
Pitch Contour of (4B) *Ani mārah besar*.

The same characteristics are sown by the adjective *marah* that occurs in a sentential environment, as illustrated in (4). Acoustically, the word *marah* gets level intonation as shown in Fig. 6. The utterance made by speaker B means that Ani is extremely angry.

**Table:** 

(4)	A: Ani marah?
	Ani angry
	‘Is Ani angry?’
	B: Ani mārah besar
	Ani angry big
	‘Ani is extremely angry’

### 4.2 Interrogative (LH) intonation

The interrogative mode of the Indonesian language is formally expressed using the question word (QW) *apakah*. This question word is employed for a yes-no question. There are five ways (5a, 5b, 5c, 5d, and 5e) for expressing interrogative sentences in Indonesian.

**Table:** 

(5)	a. Apakah dia makan?
	QW 3SG eat
	‘Is (s)he going to eat?’
	b. Apakah dia makan
	‘Is (s)he is going to eat?’
	c. Apakah dia makan?
	‘Is (s)he is going to eat?’
	d. Dia makan?
	‘Is (s)he going to eat’
	e. Makan?
	‘(Is (s)he going to) eat?

First, as seen in the (5a) example, the question word *apakah* gets the intonation that makes the whole sentence in an interrogative mode. Second, in the (5b) example, flexibility in the intonational structure is given, next to giving the question word a falling and rising intonation; a rising intonation can be placed on the verb. Third, in the (5c) example, only the word *makan* gets rising intonation and the question word does not get intonation. In the (5d) example, the question word is left out and the rising intonation is on the word *makan*, it makes the resulting sentence interpreted as a (colloquially) interrogative question. And finally (5e) is the one-word utterance that gets the rising intonation (LH) and it still encodes an interrogative mode. Importantly, with contextual support, (5e) has the same interrogative meaning as the previous four sentences. This is in line with the observation made by Ref. [12] in English words such as *guitar* and *glitter* are used as a one-word utterance (having L-H pitch contours) to denote a question.

**Fig. 7:**
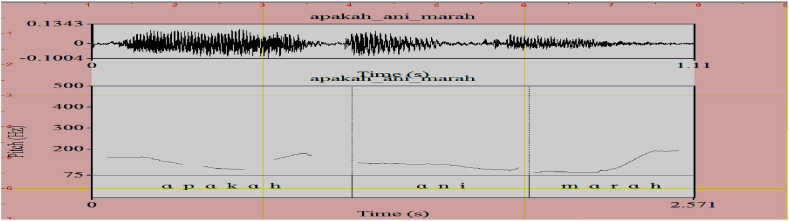
Pitch Contour of (6) *Apakah Ani marah*.

When the predicate is filled by an adjective, the intonation can fall on both the question word and the adjective predicate. Similar to the verbal predicate, for the sentence to indicate an interrogative mode, the intonation can fall on both the question word and the adjective predicate. The question word has a fall and then a rising pitch while the adjective gets a rising pitch as seen in (6) and the corresponding pitch contour in Fig. 7.

**Table:** 

(6)	Apakah Ani marah?
	QW Ani angry
	‘Is Ani angry?’

The word *marah* that occurs in isolation (7) also results in the same (interrogative) acoustic signal. This pitch contour is illustrated in Fig. 2.

**Table:** 

(7)	A: Bagaimana Ani?
	how Ani
	B: Marah?
	angry
	(Is she) angry?

### 4.3 Doubting (L) intonation

Doubting intonation is signaled by low dipping or falling-rising pitch contours. This doubting intonation is the most difficult to identify by Indonesian listeners. There are two meaning characteristic differences attributed to doubting expression in Indonesian, strong doubt and mild doubt. Strong doubt is specifically related to the situation such that the speaker is not certain of the truth/answer to the question. This is illustrated in (8). In this situation, the expression related to the person in question is negated.

**Table:** 

(8)	A: Dimana Jon?
	where John
	‘Where is John?’
	B: Tǎhu
	know
	‘I don't know’

Second, mild doubt has to do with the fact that the speaker partly agrees with the truth of the lexical item. We simply render this context as in B, i.e. the speaker doubts that Anita is beautiful.

**Table:** 

(9)	A: Anita cantik
	Anita beautiful
	‘Anita is beautiful’
	B: Cǎntik
	‘(I doubt that she is) beautiful’

While there are two slightly different nuanced meanings associated with doubt (strong and mild doubt), both show similar pitch contours. The pitch contours of *makan* ‘eat’, *tahu* ‘know, *cantik* ‘beautiful’, and *marah* ‘angry’ are the same in that they show a falling and then a rising pitch as shown in the doubting expressions in Fig. 3.

### 4.4 Neutral (HL) intonation

The term neutral intonation here relates to words when used in a complete sentence whose environment is often associated with canonical declarative clauses in which no focus questions or polar questions are found [13]. In this situation, the lexical items that get these pitch contours can be said to be atonal [27]. This situation can be illustrated in the intonational structure that operates at a sentential level as in (10).

**Fig. 8:**
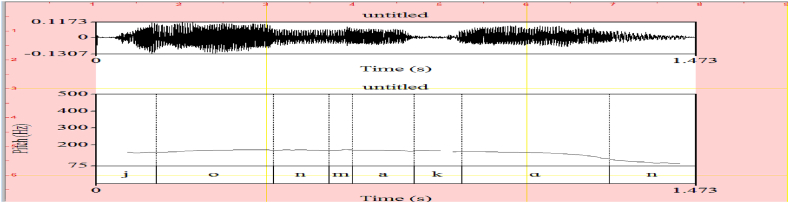
The pitch contour of (10).

The sentence *Jon makan* ‘John ate’ has two phonological phrases (PhPs) the subject constituent and the verbal constituent. In the former case, it starts with a low pitch and then gradually rises to end up with the H edge. Then, the second PhP is realized by the verbal predicate constituent which begins with the H edge and ends with the L edge. The verbal predicate gets HL or neutral intonation as indicated in Fig. 8. The meaning associated with the verb *makan* in its neutral intonation is conceptual, that is, it is the same meaning as the one found in a dictionary. It does not have any connection with pragmatically motivated interpretation.

**Table:** 

(10)	L H	H H L
	[Jon]	[makan]
	‘John ate’

It has to be noted that the rise and falling pitch contour in the neutral intonation context is not affected by the pitch of the phonological phrase that occurs immediately before it (the phonological phrase that denotes the subject). However, it occurs independently as bearing the HL pitch contour.

**Fig. 9:**
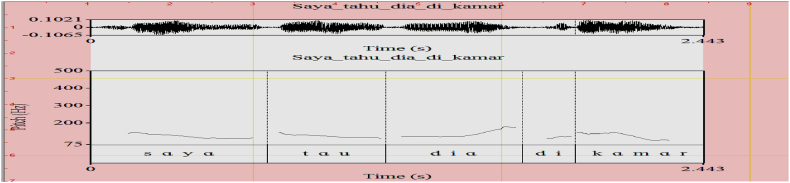
The Pitch Contour of (11) (B) *Saya tahu dia di kamar*.

Consider the prosodic structure of the utterance made by Speaker B in (11). It consists of two IPs. The first IP is realized by *saya tahu*, consisting of two PhPs. The first one begins with H pitch and ends in L pitch. The second IP has HL pitch contour filled by the verbal predicate *tahu*. The pitch pattern of the target word *tahu* is not affected by the first PhP. It stands in a neutral intonation context as shown in Fig. 9.

**Table:** 

(11)	A: Dimana Jon?
	where John
	‘Where is John?
	H L H L L H H L
	B: [Saya] [tahu] [dia ] [di kamar]
	SG know 3SG in room
	‘I know he is in the room’

#### 4.4.1 Interrogative intonation versus doubting intonation

In the current study, participants were very successful in identifying the meaning of stimulus words that are related to neutral intonation (99% correct) and emphasizing intonations (99% correct. For interrogative intonations and doubting intonation, only 96%, on average, of the listeners were able to correctly identify the different intonation contours and their associated meaning.

As has been indicated above, neutral intonation does not pose a problem for the respondents to identify the meaning of each stimulus word. This is since neutral intonation is relatively easy to identify. It directly relates to its conceptual meaning and as we know that canonical declarative clause is the basic structure of a clause that the native speakers of a language first acquire. In addition to this, the emphasizing intonation does not present any problem for the respondents/listeners either. In, every day or colloquial conversation words associated with emphasizing intonation are relatively easily recognized by Indonesian speakers because it is often used, as the name suggests, for emphasizing the quality, the condition, or the size of an entity. In doing so, the vowel segment of the first syllable of the lexicon in question is lengthened.

However, slightly more difficulty seems to be encountered by the listeners when distinguishing the interrogative intonation and the doubting intonation. This might be because the rising interrogative intonation contour has considerable similarity to the low falling rising doubting pitch contour. It is not surprising then some participants were less accurate in differentiating them. The low dipping intonation associated with doubt in the word *marah* ‘angry’ got 86% correct while the word *makan* ‘eat’ with the rising interrogative intonation got 92% correct. We might attribute this difficulty to the fact that the falling-rising or low dipping contour might be similar to rising (LH) interrogative contour and the listeners interpret it as such.

In sum, we have demonstrated that the Mandarin tonal system tied to its pitch pattern is readily mappable to the Indonesian intonational system. A question arises as to how we relate this phenomenon to Indonesian. As has been mentioned above, Indonesian has long been in contact with Mandarin Chinese by borrowing a wealth of lexical items and more importantly, Indonesian have been exposed to the Mandarin tonal system, might have borrowed it, and ultimately mapped it onto the Indonesian intonational system. While the lexical tones in Mandarin carry different meanings, the four-way Mandarin tonal contrast which has been mapped onto the Indonesian intonational system also has distinct meanings and is made distinct in their pragmatic context. We illustrate them here again with the verb *makan* ‘eat’ that can enter into the four different intonational pitches resulting in the verb's having four different meanings. (One-word utterance is here used with its appropriate contexts)

**Table:** 

(12)	T1 (emphasizing intonation) *makan* ‘(I emphasize that I want to) eat’
	T2 (interrogative intonation) *makan* ‘(Do you want to) eat?
	T3 (doubting intonation) *makan* ‘(I doubt that I want to) eat’
	T4 (neutral intonation) *makan* ‘(I want to) eat’

What is worthy of a note is that the distinction between pitch contours in the tonal system and those in the intonational system such as Indonesian seems to be the fact that pitch assignment in the tonal system is fixed in that what constitutes T1, T2, T3, and T4 has a clear place in the lexical items that have them. Thus a word belonging to T1 must have a fixed pitch contour to carry the intended meaning. A similar situation holds for Indonesian expressions except that the distinct pitch contours are associated with distinct intonational meanings (emphasizing expressions, interrogative expressions, doubting expressions, and neutral expressions).

## 5 Conclusion

The current study suggests that Mandarin four-way contrasts of pitch contours (T1, T2, T3, and T4) are available in Indonesian. This is evident from the result of the experiment which shows that when hearing stimuli with distinct pitch contours, listeners were able to correctly assign intonational meaning (emphasizing expressions, interrogative expressions, doubting expressions, and neutral expressions), with the average correct identification for all the stimulus words being 98%. The melodic signal does not only occur in a sentential level, in which the rising intonation is placed at the initial or the end of a sentence but the intonation expression can be represented by a single word, an environment in which it serves as a minor clause and it gets pitch contour transcribed as H, LH, L, or HL (emphasizing expressions, interrogative expressions, doubting expressions, and neutral expressions), respectively. The words that have a level tone (H) get the meaning of emphasizing or strong agreement. One-word intonational expressions with the LH tone have interrogative meanings. Words with a low dipping or falling rising tone (L) are interpreted as having doubting meaning. Finally, one-word intonational expressions with a falling contour have the neutral conceptual (dictionary) meaning (HL). The pitch placement in each word does not change the overall lexical meaning of those words nor does it change the categorical status of the words. Rather, the intonational meanings of the associated words depend on the context in which they occur.

The participants involved in the present study came from a specific goup (i.e. university students). Future studies should recruit participants coming from a variety of demographic background to gain more valid findings.

The knowledge derived from the present study might be significant for LOTE (languages other than English) education. Needless to say, the findings of this study will undoubtedly benefit those involved in Indonesian language teaching. Since all the pitch contours gain currency in Indonesian, it is recommended that Indonesian language teachers might need to incorporate into their pedagogical practice not only neutral intonation (corresponding to T4) and interrogative intonation (corresponding to T2) which tend to occur more in daily conversation. Indonesian language teachers might also need to introduce emphasizing intonation (corresponding to T1) and doubting intonation (corresponding to T3) in their teaching materials more extensively.

## Author contribution statement

I Nyoman Udayana: Conceived and designed the experiments; Analyzed and interpreted the data; Wrote the paper.

I Nyoman Aryawibawa: Conceived and designed the experiments; Analyzed and interpreted the data.

I Nyoman Sedeng: Conceived and designed the experiments; Performed the experiments.

Joan A. Sereno: Conceived and designed the experiments; Analyzed and interpreted the data.

## Funding statement

This research was funded by PKLNU (Udayana University International Reserach Collaboration) Scheme with Research Implementation Assignment Agreement Number: B/96-106/UN14.4.A/PT.01.05/2021.

## Data availability statement

Data will be made available on request.

### Declaration of interest's statement

The authors declare no competing interests.

## Figures and Tables

**Table:** 

/makan/	Mean (F_0_)	Min	Max
/m/	120.68	119.03	122.78
/ɑ/	122.32	119.58	123.25
/k/	131.44	123.09	135.39
/ɑ/	121.99	119.5	132.44
/n/	119.01	117.97	119.59
Makan	121.85	117.97	135.39

**Table:** 

/tau/	Mean (F_0_)	Min	Max
/t/	119.28	119.13	119.55
/ɑ/	116.65	113.97	119.97
/u/	121.83	118.39	123.8
Tahu	118.32	113.97	123.8

**Table:** 

/cantik/	Mean (F_0_)	Min	Max
/c/	130.19	129.48	131.5
/ɑ/	127.04	125.28	131.3
/n/	125.59	121.31	126.96
/t/	125.22	123.32	126.24
/i/	121.98	116.96	126
/k/	116.36	116.07	116.96
Cantik	124.56	116.07	131.5

**Table:** 

/marah/	Mean (F_0_)	Min	Max
/m/	122.95	121.4	123.9
/a/	122.53	120.41	124.14
/r/	122.01	119.84	124.2
/ɑ/	120.72	108.93	123.87
/h/	108.82	102.94	113.26
Marah	120.75	102.94	124.2

**Table:** 

/makan/	Mean (F_0_)	Min	Max
/m/	107.27	106.37	109.11
/ɑ/	111.49	109.11	112.75
/k/	111,78	109,24	120,36
/ɑ/	160.94	120.36	212.14
/n/	237.59	212.14	251.05
Makan	166,66	106.37	251.05

**Table:** 

/tau/	Mean (F_0_)	Min	Max
/t/	113.64	113.61	113.69
/ɑ/	115.77	111.89	133.07
/u/	190.93	133.07	244.38
Tahu	153.58	111.89	244.38

**Table:** 

/cantik/	Mean (F_0_)	Min	Max
/c/	116.77	116.51	117.23
/ɑ/	104.02	108.03	117.03
/n/	108.78	106,09	109.84
/t/	113.02	106.6	116.01
/i/	155.19	107.85	239.31
/k/	240.76	238,00	244.44
Cantik	145.31	106,09	244.44

**Table:** 

/marah/	Mean (F_0_)	Min	Max
/m/	117.04	114.75	118.53
/a/	107.87	101,93	114.75
/r/	97.81	96.54	102.19
/ɑ/	154.01	96.59	250.09
/h/	251.1	250.09	252.22
Marah	135.53	96.54	252.22

**Table:** 

/makan/	Mean (F_0_)	Min	Max
/m/	133.92	130.75	138.61
/ɑ/	128.69	104.62	147.51
/k/	115.67	113.59	117.64
/ɑ/	146.43	111.9	208.48
/n/	224.83	208.48	242.99
Makan	141.23	104.62	242.99

**Table:** 

/tau/	Mean (F_0_)	Min	Max
/t/	145.15	142.24	147.74
/ɑ/	128.1	98.98	144.43
/u/	124.2	96.68	216.09
Tahu	126.77	96.68	216.09

**Table:** 

/cantik/	Mean (F_0_)	Min	Max
/c/	133.76	133.44	134.19
/ɑ/	124.67	109.91	133.58
/n/	107.65	105.44	109.91
/t/	102.67	99.01	105.58
/i/	134.14	104.26	204.35
/k/	205.57	204.35	206.11
Cantik	126.62	99.01	206.11

**Table:** 

/marah/	Mean (F_0_)	Min	Max
/m/	201.03	200.39	202.57
/a/	167.48	114.71	205.66
/r/	114.65	111.82	116.65
/ɑ/	177.89	116.65	251.02
/h/	259.7	251.02	271.05
Marah	169.83	111.82	271.05

**Table:** 

/makan/	Mean (F_0_)	Min	Max
/m/	146.59	146.16	147.04
/ɑ/	139.55	134.27	146.16
/k/	137.63	136.35	138.03
/ɑ/	123.11	105.77	138.03
/n/	102.25	99.37	105.77
Makan	130.11	99.37	147.04

**Table:** 

/tau/	Mean (F_0_)	Min	Max
/t/	137.34	137.34	137.34
/ɑ/	120.39	102.37	137.34
/u/	95.02	90.91	102.37
Tahu	110.54	90.91	137.34

**Table:** 

/cantik/	Mean (F_0_)	Min	Max
/c/	155.67	154.21	157.21
/ɑ/	155.61	153.36	158.17
/n/	148.49	141.05	156.97
/t/	139.00	136.54	140.32
/i/	117.46	103.35	136.54
/k/	105.28	103.20	106.15
Cantik	133.00	103.20	158.17

**Table:** 

/marah/	Mean (F_0_)	Min	Max
/m/	126.37	126.05	127.11
/a/	128.64	120.45	134.21
/r/	116.71	107.83	124.05
/ɑ/	104.85	94.91	108.42
/h/	93.35	92.56	95.31
Marah	116.86	92.56	134.21

## Footnotes

1 The verb *tahu* ‘know’ and the noun *tahu* ‘tofu’ are homographs but are phonetically distinct. Phonetically the [h] in the verb *tahu* is silent (while it is pronounced in the noun *tahu)*.
